# Mitochondrial quality control in diabetic cardiomyopathy: from molecular mechanisms to therapeutic strategies

**DOI:** 10.7150/ijbs.75402

**Published:** 2022-08-15

**Authors:** Chen Cai, Feng Wu, Jing He, Yaoyuan Zhang, Nengxian Shi, Xiaojie Peng, Qing Ou, Ziying Li, Xiaoqing Jiang, Jiankai Zhong, Ying Tan

**Affiliations:** 1 Department of Critical Care Medicine, Nanfang Hospital, Southern Medical University, Guangzhou 510515, China.; 2 Department of Critical Care Medicine, The First School of Clinical Medicine, Southern Medical University, Guangzhou 510515, China.; 3 Department of Cardiology, Shunde Hospital, Southern Medical University (The First People's Hospital of Shunde), Foshan 528308, Guangdong, China.

**Keywords:** Diabetic cardiomyopathy, mitochondrial quality control, mitochondrial fission, mitochondrial fusion, mitophagy

## Abstract

In diabetic cardiomyopathy (DCM), a major diabetic complication, the myocardium is structurally and functionally altered without evidence of coronary artery disease, hypertension or valvular disease. Although numerous anti-diabetic drugs have been applied clinically, specific medicines to prevent DCM progression are unavailable, so the prognosis of DCM remains poor. Mitochondrial ATP production maintains the energetic requirements of cardiomyocytes, whereas mitochondrial dysfunction can induce or aggravate DCM by promoting oxidative stress, dysregulated calcium homeostasis, metabolic reprogramming, abnormal intracellular signaling and mitochondrial apoptosis in cardiomyocytes. In response to mitochondrial dysfunction, the mitochondrial quality control (MQC) system (including mitochondrial fission, fusion, and mitophagy) is activated to repair damaged mitochondria. Physiological mitochondrial fission fragments the network to isolate damaged mitochondria. Mitophagy then allows dysfunctional mitochondria to be engulfed by autophagosomes and degraded in lysosomes. However, abnormal MQC results in excessive mitochondrial fission, impaired mitochondrial fusion and delayed mitophagy, causing fragmented mitochondria to accumulate in cardiomyocytes. In this review, we summarize the molecular mechanisms of MQC and discuss how pathological MQC contributes to DCM development. We then present promising therapeutic approaches to improve MQC and prevent DCM progression.

## Introduction

### Epidemiology and predisposing factors of diabetic cardiomyopathy (DCM)

**Figure 1:**
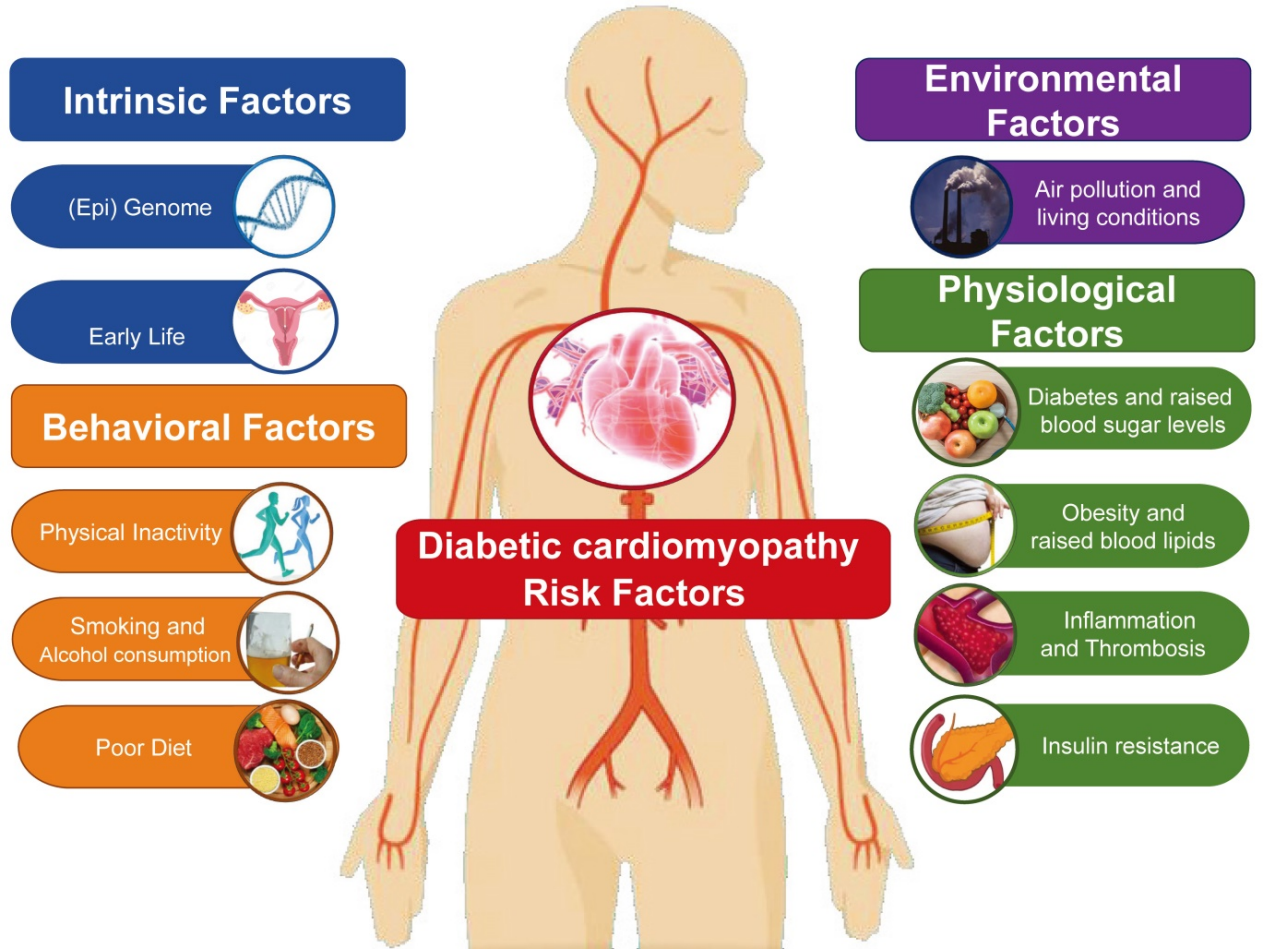
The contribution of altered metabolism to cardiovascular risk.

From a pathophysiological viewpoint, DCM is a heart failure state primarily attributable to chronic myocardial metabolic disorder (Table 1). Clinically, myocardial damage etiology is always labeled as coronary artery disease (such as angina and myocardial infarction) in light of the irreplaceable role served by coronary arteries in managing heart blood flow 1. However, some patients (especially middle-aged women) with no evidence of coronary artery disease detectable with imaging tools or functional tests may suffer from angina and myocardial infarction as a result of cardiac microvascular spasm or endothelial dysfunction 2. In addition, several diseases of the heart muscle, including hypertrophic cardiomyopathy and arrhythmia-induced cardiomyopathy, clinically present with ischemia-related symptoms such as angina or ventricular wall motion abnormalities due to increased myocardial oxygen demand 3. Consequently, the etiology of DCM in an individual patient is rarely unambiguous in practice and is frequently complicated by overlap between a classical ischemic component and discernable non-ischemic causes such as viral myocarditis, hypertension, diabetes, valvular disease, dyslipidemia, obesity, and unhealthy lifestyles 4 (Figure 1). Although research into the pathological mechanism of DCM is ongoing, we have not yet precisely defined its epidemiology and predisposing factors. Our aim, therefore, is to provide reference for the prevention of such diseases by reviewing the risk factors that can induce DCM or the pathological mechanism underlying DCM.

DCM affects more than 23 million people worldwide, including more than 15.5 million people in the United States 5. Its prevalence in women (5%) is lower than in men (7.5%), and older women with DCM are more likely to have atypical ischemic heart disease symptoms such as a coronary microcirculation disorder, coronary microvascular injury, coronary artery dissection, or Takotsubo cardiomyopathy 6, 7. In the United States, 6.6 million women are diagnosed with DCM each year, including 2.7 million patients with a history of myocardial infarction 8. In the Framingham study, men and women with coronary heart disease were followed for up to 44 years 9.The results show that 40-year-old men have a higher lifetime risk of coronary heart disease than women. However, the incidence among women aged 65-94 is higher than among those aged 35-64; that is, that rates of morbidity and mortality due to coronary artery disease are higher among postmenopausal women 10. Among the numerous other risk factors for DCM are obesity, hypertension, hyperglycemia, fat metabolism disorders, atherosclerosis, sedentary lifestyle, lack of exercise, smoking, and pressure 11. These factors may interact with each other and directly or indirectly lead to the onset of DCM or affect the process of disease 12.

### Age

Age is a key risk factor positively correlated with the prevalence of DCM 13. This in large part reflects the increased incidence disorders in lipid metabolism and hyperlipidemia seen with increasing age. With declines in organ function, blood lipid metabolism is slowed, leading to hyperlipidemia and diabetes, which may be accompanied by other ailments such as hypertension 14, 15. Such metabolic disorders can exacerbate coronary deposition of lipid plaque, which can progress to coronary atherosclerosis or microvascular damage, indirectly leading to the occurrence of DCM 16. Although age may be related to the occurrence of DCM, it is not an inevitable factor. Men over 45 years of age and women over 55 years of age have similar increased risk of DCM. Moreover, the DCM mortality rate among women aged 35 to 44 is higher than among men of the same age 17.The reason may be that there is a lack of knowledge about DCM-inducing factors, including the roles of blood sugar, blood fat and body weight. Obesity, work stress, smoking, sedentary lifestyle, lack of exercise and metabolic syndrome are considered to be the arch criminals leading to DCM 18, 19. Age is only one factor contributing to the pathogenesis of DCM. Indeed, DCM can even occur in some younger patients, including patients under 20 years old. In those cases, genetic factors and family genetic history play key roles. For example, in cases of early-onset cardiomyopathy, comorbidities involving abnormal blood lipid metabolism are often present.

### Genetic factors

Cardiovascular disease is one of more than 3000 diseases in which genetic factors play a role 20. Studies have shown that patients with coronary heart disease, hypertension, and other cardiovascular diseases, are more than four times more likely to die of similar diseases than the general population. If the parents of monozygotic twins suffer from cardiovascular disease, the probability of cardiovascular disease in both twins is more than six times higher than in the general population. DCM is a complex and multifactorial cardiovascular disease and it, too, is affected by genetic as well as environmental factors 21, 22.

Coronary atherosclerosis is the main cause of coronary artery stenosis and DCM. Low density lipoprotein (LDL) cholesterol molecules contribute to the development and progression of atherosclerosis because it is the main type of lipid absorbed by cell within atherosclerotic lesion. Therefore, genetic changes affecting plasma LDL levels are an important factor in the development of DCM 23. Familial hypercholesterolemia caused by genetic factors will be accompanied by high levels of LDL and cholesterol, which accelerates development of atherosclerosis and coronary heart disease 24-26. Nonetheless, the development of DCM still mainly depends on an individual's health behavior. Without the influence of genetic factors, a high salt diet, high-fat diet, long periods sitting and other stressful lifestyle or working environment factors will also lead to the onset of DCM 27.

### Smoking

Smoking is considered to be an independent cause of DCM. It is obviously related to the onset of DCM and coronary heart disease 28, 29. Studies have found that smokers are 70% more likely to develop DCM than non-smokers. Carbon monoxide, thromboxane, nicotine and other harmful components in cigarette smoke adversely affect the patient's vasodilator function and then cause vascular dysfunction and damage to the vascular endothelium 30, 31. Nicotine in cigarettes can also cause thickening and abnormal contraction of blood vessel walls. In addition, increased levels of cadmium and lead in the blood of smokers can induce oxidative damage to the vascular endothelial structure, reducing the oxygen supply of myocardial cells while increasing oxygen consumption by myocardial cells, leading to myocardial hypoxia 32.

Smoking also promotes abnormal lipid metabolism, aggravating atherosclerosis. Nicotine promotes oxidation of LDL, after which the oxidized LDL bind to receptors on endothelial cell surfaces and form lipid plaques over time 33. Vascular endothelial dysfunction caused by smoking can also promote the release of inflammatory factors and increase the secretion of prostaglandin F2α metabolites into the circulation, promoting the development of atherosclerosis 34, 35. In patients with acute myocardial infarction, smoking can increase levels of thrombolysis and expression of inflammatory factors, which will aggravate myocardial ischemia directly related to smoking 36, 37. Smoking cessation reduces the number of serum inflammatory markers, and the longer the patient goes without smoking, the more obvious the effect will be.

### Diet

Both epidemiological and experimental results show that high intake of animal fat and cholesterol is positively correlated with the incidence of DCM 38. High cholesterol and triglyceride caused by diets high in fat, calories, salt and sugar can contribute to the development hyperlipidemia and DCM as well as hypertension 39, 40. A healthy diet is a primary factor contributing to DCM prevention. The benefits of the Mediterranean diet are related to the reduction various cardiovascular risk factors, including inflammation, vascular endothelial injury, insulin resistance and especially the formation of atherosclerosis and arterial stenosis 41. Importantly, the benefits of a healthy diet are not limited to DCM, and a good diet should come first in any effort to reduce cardiovascular risk.

### Mental stress

Mental stress is a major risk factor for DCM, though the mechanism by which stress contributes to the pathogenesis of coronary atherosclerosis or DCM has not yet been confirmed 42, 43. Nonetheless, clinical studies have shown that mental stress can be the main factor underlying increases in the prevalence of DCM and the triggering factor for acute myocardial ischemia (as indicated by sudden death, myocardial infarction, ST segment depression) in patients with coronary atherosclerosis 44, 45.

In a laboratory environment, mental stress can cause myocardial ischemia in some patients. The prevalence of myocardial ischemia caused by mental stress depends on the stressor used, the patient group, and especially the diagnostic tool. In fact, ischemic attacks caused by mental stress are usually “silent,” and their severity and scope are smaller than those caused by exercise stress tests. Patients with myocardial ischemia caused by mental stress tend to have higher scores in terms of aggression, anger, and hostility. These psychological characteristics are related to higher cardiovascular responsiveness. After being under tremendous pressure, the heart rate and blood pressure will increase rapidly, and the increase is greater. The mechanism of the myocardial ischemia induced by mental stress may be an increase in myocardial oxygen demand due to the increased heart rate and blood pressure 46, 47. It is noteworthy that psychological ailments such as depression and anxiety can lead to or affect the condition of patients with DCM 48. Depression can directly affect the mortality rate among patients with DCM 49, 50. Attention should therefore be paid to the evaluation and treatment of psychological factors in patients with DCM.

### Obesity

Obesity is generally considered to be a risk factor for DCM, in part because a high-calorie/fat diet contributes to the progression of atherosclerosis. Epidemiological studies now tend to support this argument. However, angiographic studies have shown little or no correlation between total fat mass and coronary atherosclerosis 51. Obesity, especially centripetal obesity, is closely related to the traditional risk factors of DCM and dyslipidemia. This may be due to the high homo-cysteinemia, high lipoprotein levels and increased thrombosis in obese people. Blood lipids are often beyond the standard range in obese individuals, and high calorie diets lead to elevations in LDL, triglycerides and blood pressure, thereby promoting the formation and/or progression of coronary atherosclerosis 52, 53. Fat in obese people often accumulates within important organs and blood vessels, which leads to abnormal glucose and lipid metabolism, increases in blood pressure, myocardial load, and myocardial oxygen consumption while increasing body weight. Metabolic processes are greatly altered within cardiomyocytes due elevated sugar or calorie sources and low self-consumption rate. The resultant dysregulation of fat and glucose metabolism can lead to an imbalance in mitochondrial oxidative phosphorylation and energy metabolism and serious myocardial injury.

In addition to increasing myocardial load and dyslipidemia, obesity has adverse effects on coronary circulation. These include coronary vasomotor dysfunction and coronary artery occlusion 54.Obesity associated with dyslipidemia easily leads to increases in coronary and microvascular resistance, which is the primary factor contributing to the pathogenesis of coronary microvascular disease. Moreover, adipose tissue with infiltrating macrophages in obese patients are a key source of pro-inflammatory mediators, which can induce microvascular inflammation and myocardial hypoperfusion and further release of pro-inflammatory mediators into the coronary circulation 55, 56. These mediators impair coronary microcirculation and are a main cause of DCM and heart failure in obese patients. More importantly, in addition to their underlying diseases, obese people are often sedentary, eat an unhealthy diet, and engage in other bad habits. The interaction among these habits hinders the formation of coronary collateral circulation 57. In addition, obese people often exhibit insulin resistance or type 2 diabetes as well as hyperlipidemia and hyper-fibrinogen 58, 59, all of which are predisposing factors for atherosclerosis and arterial stenosis and, ultimately, DCM.

### Sedentary lifestyle

Many studies suggest that a sedentary lifestyle can increase the risk of coronary atherosclerosis or DCM 60. The rapid development of modern science and technology has changed people's daily live and commuting, resulting in a significant decline in people's activity level. Many office workers and the elderly now have a sedentary lifestyle characterized by long periods of inactivity. This lack of activity/exercise can lead to obesity, hypertension, arrhythmia, atherosclerosis, slowed blood flow, and thrombosis 61, 62. The coronary atherosclerosis or coronary artery stenosis that often develops as a result of this lifestyle becomes the main cause of DCM. However, interventions that reduce sedentary behavior may improve cardiovascular health and well-being.

## Molecular mechanisms of mitochondrial dysfunction in DCM

### Mechanisms of DCM

DCM is a special cardiac complication characterized by chronic cardiomyopathy caused by diabetes 10. The pathological process includes oxidative stress, energy metabolism, inflammatory reaction and increased myocardial cell apoptosis in cardiac myocytes 10, 63 (Table 2). Cardiac manifestations include early diastolic dysfunction, cardiac hypertrophy, ventricular dilatation and systolic dysfunction, which eventually lead to heart failure 64. In diabetic mice, studies have showed the evidence of impaired mitochondrial function in heart tissue, which is related to mitochondrial ultrastructural defects 65. Insulin resistance leads to ROS overproduction in cardiomyocytes 66. Studies have also shown that hyperglycemia can lead to the fragmentation of mitochondria in cardiomyocytes, induce mitochondrial division, and produce mitochondrial ROS 67. Additionally, high glucose can induce mitochondrial division in cardiomyocytes due to an increase in the o-glcnacylation of Drp1 and the decrease of Drp1 in Ser637 phosphorylation. In addition, high glucose decreased the content of OPA1 and augmented its glycosylation. Increasing OPA1 protein level or decreasing OPA1 glycosylation could block hyperglycemia-related mitochondrial division. Clinical trials show that antagonizing ROS by antioxidants alone is not enough to attenuate DCM 68, 69. A more effective strategy is to improve the overall ability of mitochondrial quality control to maintain healthy mitochondrial pools needed to support cardiac contractility.

### Mechanisms of mitochondrial dysfunction

In recent years, the prevalence and mortality of cardiovascular diseases, such as atherosclerosis, hypertension, myocardial hypertrophy and diabetic cardiomyopathy, are increasing all over the world, which are the main causes of death and disability. Mitochondria are organelles with double membrane structure, which can meet the high energy requirement of heart metabolism through oxidative phosphorylation 70-72. Mitochondria are extremely sensitive to the changes of their environment. When the external environment, such as impaired nutrition provision and decreased oxygen supply, mitochondria are able to make corresponding metabolic adaptation 73. However, these protective mitochondrial adaptations, including mitochondrial dynamics regulation, mitophagy activation, mitochondrial biogenesis augmentation, mitochondrial bioenergetics improvement and mitochondrial anti-oxidative capacity intensification are usually impaired in many cardiovascular diseases 74, which is therefore accompanied by mitochondrial respiratory chain dysfunction, ATP synthesis disorder, oxidative stress and mitochondrial integrity loss 75, 76. In dysfunctional mitochondria, the decoupling of electron transport chain leads to ROS production and ATP depletion, causing extensive damage to cardiomyocytes and activating cell apoptosis or necrosis 77. At present, the relationship between mitochondrial dysfunction and cardiovascular diseases has been confirmed 78. Among them, abnormal mitochondrial dynamics and impaired mitochondrial function have been found to be closely associated with decreased mitochondrial biosynthesis, increased mitochondria lipid oxidative damage, and excessive mitochondrial DNA breakage 79-81; these alterations have been identified as the pathophysiological basis of a variety of cardiovascular diseases.

Mitochondrion is the power source of cells. ATP is synthesized by the metabolites of fatty acids, glucose and amino acids 82, 83; these alterations are primarily occurred in mitochondria 84. In addition, mitochondria play an important role in calcium homeostasis 85, hemoglobin synthesis, fatty acid oxidation, ROS production and clearance, and cell growth and apoptosis regulation 86, 87. Therefore, the normal structure and function of mitochondria have a role in meeting the energy requirements of vital activities, maintaining the homeostasis of cellular environment and regulating cell growth.

Mitochondrial dysfunction refers to the structural and functional abnormalities of mitochondria caused by the accumulation of ROS in cells under the stimulation of various damage factors, such as ischemia and hypoxia 88. The features of mitochondrial dysfunction include a decrease of membrane potential, a reduction in mitochondrial biosynthesis, a drop in ATP synthesis and a damage to mitochondrial respiratory chain. Besides, the well-known outcome of mitochondrial dysfunction is ROS overproduction at the mitochondrial respiratory chain, causing oxidative damage to cell and mitochondrial protein, lipid and DNA 89. Small ROS production will further augment mitochondria damage and thus promote mitochondrial ROS production, which forms a vicious circle of mitochondrial damage. Once the ROS production exceeds the scavenging capacity of antioxidant system, cellular oxidative stress and tissue damage occur. It is necessary to point out that most of the ROS in the heart are produced by the decoupling of mitochondrial respiratory chain.

## Pathophysiology of mitochondrial quality control (MQC) in DCM

### Mitochondrial fusion and fission dynamics

**Figure 2:**
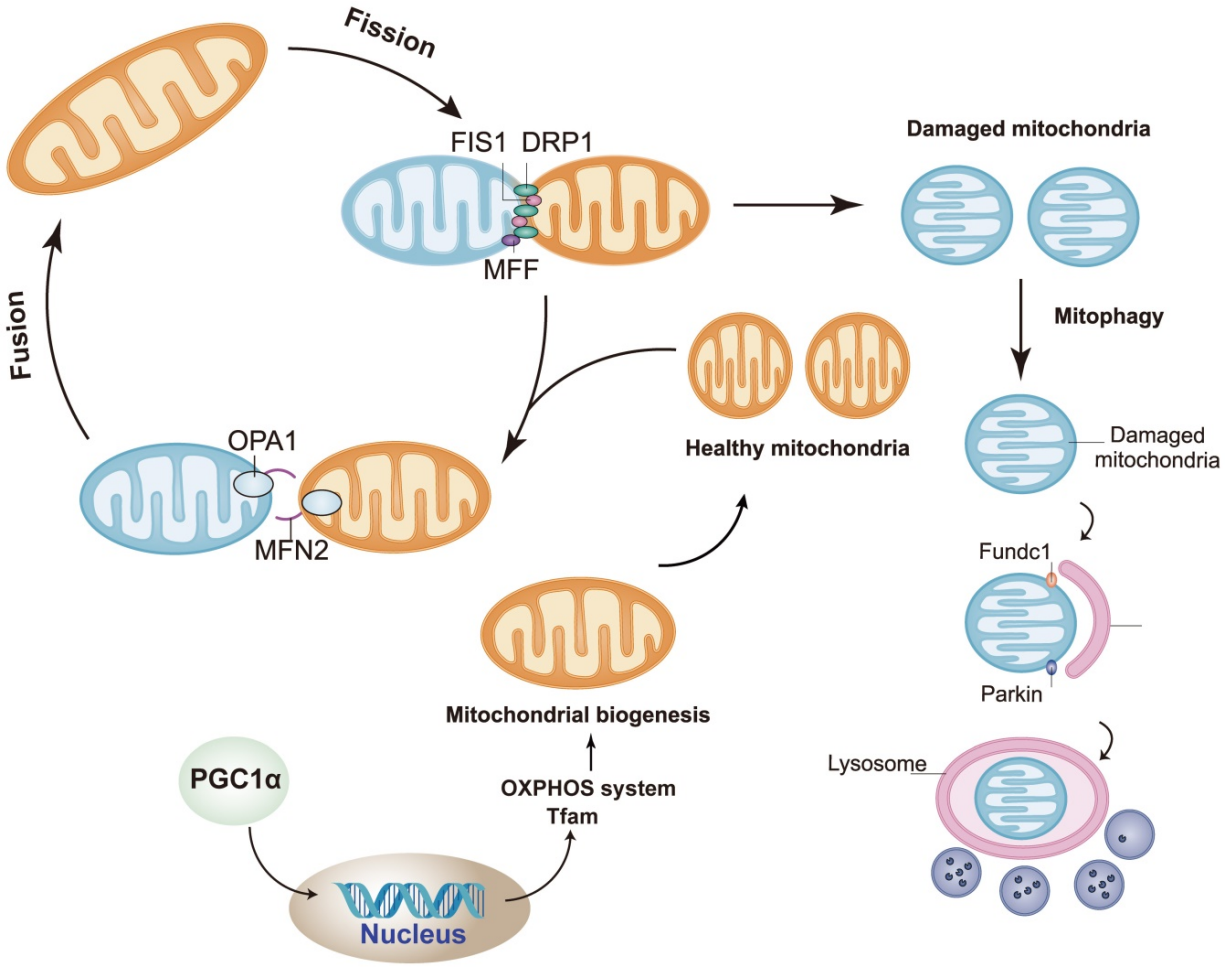
The regulation of mitochondrial dynamics. Mitochondrial fusion protein Mfn1/2 promotes mitochondrial outer membrane fusion whereas OPA1 promotes fusion of inter membrane of mitochondria. The regulatory factors of mitochondrial division are Drp1, Mff, Fis1, Mid49 and Mid51. (By Figdraw (www.figdraw.com)).

Mitochondria are highly dynamic organelles in most mammalian cells. Through continuous fusion and division, the shape, size and quantity of mitochondria can be changed to meet the metabolic needs of cells (Figure 2) 66, 90. In mitochondrial dynamics, the regulation of mitochondrial division and fusion is a group of dynamic-related GTP enzymes 91, including fusion of mitochondrial endocardium and mitochondrial outer membrane. Mitochondrial fusion protein Mfn1/2 promotes mitochondrial outer membrane fusion whereas OPA1 promotes fusion of inter membrane of mitochondria 92. Mammalian Mfn1/2 is a highly similar protein (human similarity is about 80%), which is composed of 737 and 757 amino acid sequences, respectively 93. They have homology, about 80% similar structure related sequences, and have homogeneity and heterogeneity in physical function. They will form Mfn1 homopolymer, Mfn2 homopolymer and Mfn1/2 heteropolymer. The second component regulating mitochondrial fusion is OPA1, a transmembrane protein closely related to mitochondrial inner membrane, which is expressed in a variety of variants through alternative splicing and post-translational proteolysis, resulting in short (s) and long (L) subtypes. Deletion or mutation of any of these genes will lead to embryo death and mitochondrial dysfunction. The depletion of Mfn1 and/or Mfn2 in cells leads to poor cell growth, and the decrease of cell respiration due to decreased mitochondrial membrane potential. Knockout of Mfn1 or Mfn2 is also associated with embryo death due to placental defects 94. OPA1 deficiency was characterized by mitochondrial fragmentation, decreased cristae and oxidative phosphorylation 95. The regulatory factors of mitochondrial division are Drp1, Mff, Fis1, Mid49 and Mid51. Drp1-dependent mitosis can be divided into four steps 96-99: translocation of Drp1 to the outer membrane of mitochondria, subsequent high-level assembly, hydrolysis of GTP, and final disassembly. Drp1 is an 80 kDa dynein GTPase superfamily protein 100, 101. It mainly exists in the cytoplasm in the form of dimer/tetramer, shuttling between the cytoplasm and mitochondria. The recruitment of Drp1 from the cytoplasm to the outer membrane of mitochondria is an important step in mitochondrial division. Recruitment from cytoplasm to mitochondria is mediated by several outer mitochondrial membrane proteins, including Mff, Fis1, Mid49 and Mid51 96, 102. Drp1 is recruited into mitochondria through a receptor anchored on the outer membrane of mitochondria 103. Once recruited, Drp1 further assembles around the mitochondrial tubules to form an oligomeric ring, which contracts and splits mitochondria in a GTP dependent process 104. Mff is an important factor in the recruitment of Drp1 during mitosis, and its overexpression leads to increased mitosis 105. In contrast, other drp1 receptors, Mid49 and Mid51, seem to recruit inactive forms of drp1 because their overexpression inhibits mitochondrial division 106.

### Mitochondrial fusion and fission dynamics in DCM

Mitochondrial morphological changes in cardiomyocytes were observed in *ob/ob* mice 107. In these mice, mitochondria appeared as abnormally large 'mega-mitochondria' 107. Cardiomyocytes from neonatal rats exposed prenatally to diabetes or a high-fat diet exhibited impaired mitochondrial dynamics, which resulted in shorter, wider mitochondria than those from control rats 108. These hyperglycemia-induced changes in mitochondrial dynamics seemed to be gender-specific; in fact, the male hearts contained post-translational modifications known to impair mitochondrial dynamics 108.

Another study demonstrated that Drp1 was significantly upregulated in cardiomyocytes during the progression of DCM, whereas Mfn1 and 2 were markedly downregulated 109. Genetic ablation of *Drp1* was found to protect the heart against hyperglycemic damage by sustaining cardiomyocyte viability and function 110. In diabetic hearts, Drp1 phosphorylation at Ser616 was induced, and this alteration was followed by cardiomyocyte hypertrophy and mitochondrial dysfunction, suggesting that post-translational modifications of mitochondrial dynamics proteins may contribute to mitochondrial dysfunction during DCM 111. In the setting of DCM, other phosphorylated forms of Drp1 have also been detected, such as p-Drp1^Ser637^ 112, p-Drp1^Ser579^ 113 and p-Drp1^Ser600^ 113, 114. Moreover, increased O-GlcNAcylation of Drp1 at threonine 585 and 586 was observed in diabetic heart mitochondria, correlating with elevated mitochondrial fragmentation in cardiomyocytes 115. Interestingly, lipid overload during diabetes was associated with Drp1 acetylation at lysine 642, which then promoted mitochondrial fission and cardiomyocyte death 116. In contrast, transfection of a nonacetylated Drp1 mutant (K642R) prevented hyperglycemia-induced cardiomyocyte hypertrophy and dysfunction 116, 117.

Regarding mitochondrial fusion, an *in vitro* model of DCM (primary cultured neonatal rat cardiomyocytes treated with high glucose) exhibited elevated Mfn1/2 ubiquitination, a reduced mitochondrial membrane potential, increased mPTP opening and diminished ATP production 118. Hyperglycemia also induced O-GlcNAcylation of OPA1 in neonatal cardiac myocytes, thus reducing the mitochondrial length and suppressing complex IV activity 119. Of note, a fructose-rich diet stimulated mitochondrial fragmentation in the heart by suppressing Mfn2 and inducing Drp1 without influencing OPA1 120, suggesting that different kinds of sugar may distinctly impact the regulators of mitochondrial dynamics in diabetic hearts.

Several upstream signals of abnormal mitochondrial dynamics have been described in the setting of DCM, including translocase of outer mitochondrial membrane 70 (Tom70) 121, cyclin C 122, sirtuin 1 (Sirt1) 123, estrogen 124, Gp78 125, insulin 126 and norepinephrine 127. The molecular effects of these upstream signals on mitochondrial dynamics during DCM are detailed in Table 3. In turn, the activation of mitochondrial fission in hyperglycemia-treated cardiomyocytes has multiple downstream effects, including cardiomyocyte apoptosis, oxidative stress, myocardial fibrosis 128, mitochondrial membrane potential reduction, insulin pathway deactivation, insulin resistance 129, delayed mitochondrial respiration and mitochondrial calcium overload 130. Together, these alterations may eventually induce mitochondrial dysfunction and reduce cardiomyocyte viability, accelerating the development of DCM.

### Mitophagy

**Figure 3:**
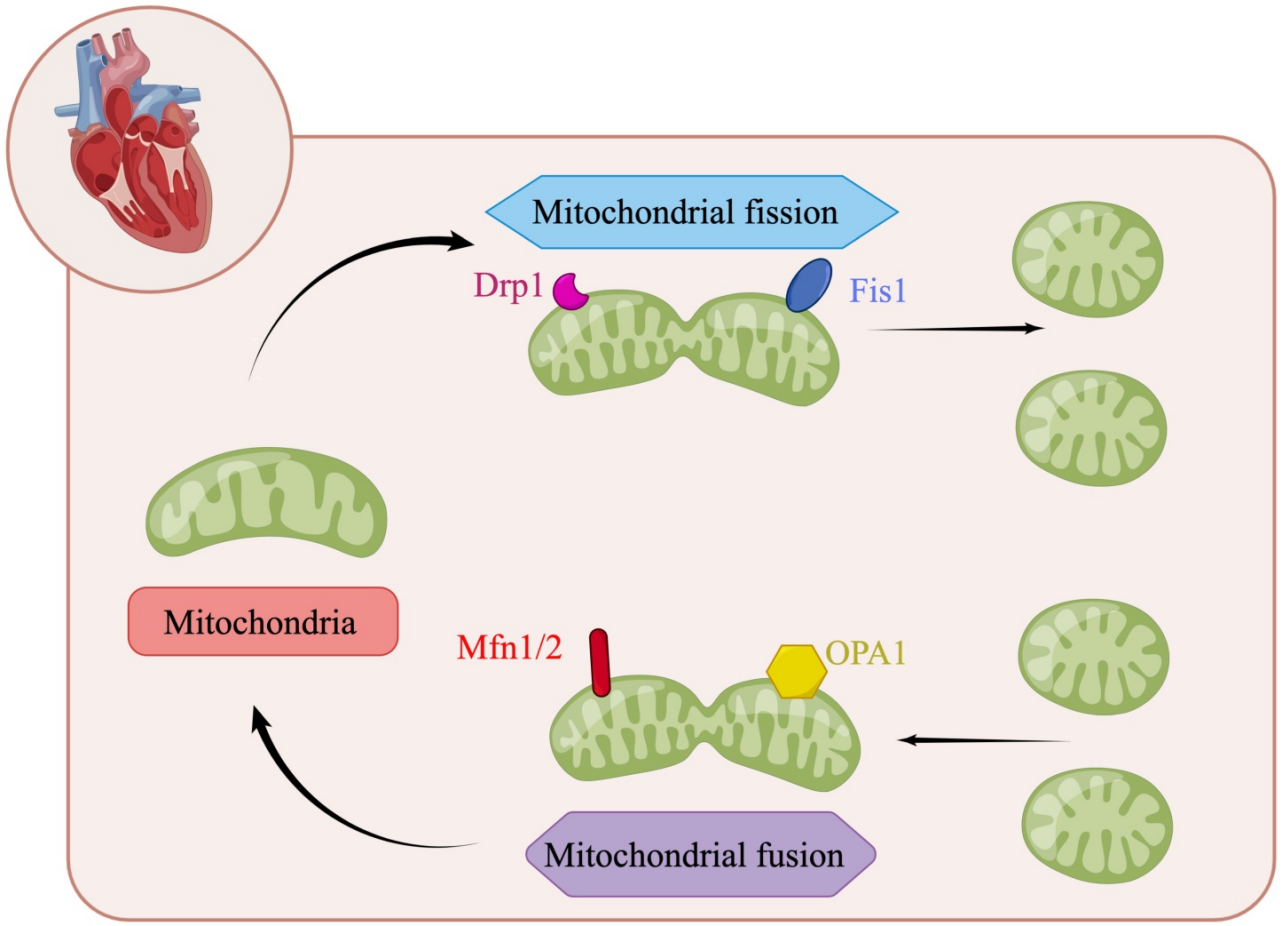
Mitophagy is a process in which autophagosomes selectively target to phagocytize dysfunctional or damaged mitochondria and transfer them to lysosomes for cell recycling (by Figdraw (www.figdraw.com)).

Mitophagy is a process in which autophagosomes selectively target to phagocytize dysfunctional or damaged mitochondria and transfer them to lysosomes for cell recycling (Figure 3) 131, 132. The division of mitochondria is considered by biologists to be mainly to separate the mitochondria of daughter cells with damaged membrane potential 133, 134. The daughter mitochondria with normal membrane potential can fuse with other mitochondria 135. The stability of mitochondrial internal environment requires a perfect balance between mitochondrial phagocytosis and mitochondrial biogenesis 136, 137. Mitophagy recognizes target mitochondria through LC3 adaptor through the ubiquitin-dependent and -independent pathways 138. The first step of ubiquitin-dependent mitophagy is the ubiquitination of mitochondrial substrate, which is the recognized by LC3 adapter 139, 140. At present, the most studied mitophagy pathway in mammals is regulated by the putative kinases such as PINK1 and Parkin, which is under the control of phosphatase and tensin homolog PTEN 141. PINK1 is a sensor of mitochondrial polarization. In healthy polarized mitochondria, PINK1 is introduced into mitochondria by transporter outer membrane complex and into mitochondria by transporter inner membrane complex. Under normal conditions, the basal PINK1 level was maintained at a low level 142. In the process of mitochondrial depolarization, the decrease of mitochondrial membrane potential is related to the accumulation of PINK1 on the outer membrane of mitochondria, which, together with ubiquitin ligase Parkin, controls the elimination of defective mitochondria 143. In addition, PINK1 can phosphorylate the Ser65 site of ubiquitin like (UBL) domain, activate the E3 ligase activity of Parkin and promote the recruitment of Parkin in the outer membrane of mitochondria 144, 145. There are many Parkin substrates on the mitochondrial outer membrane, including mitochondrial fusion protein Mfn1/2, mitochondrial outer membrane translocator, mitochondrial transporter and voltage dependent anion channel VDAC 146. By interacting with LC3 aptamer on the membrane, autophagosomes were recruited to selectively encapsulate the modified receptor mitochondria, and finally the damaged mitochondria were transferred to lysosomes for degradation. LC3 adaptor proteins include p62, NBR1, OPTN, NDP53 and TAX1BP1, but only OPTN and NDP52 are considered as their main substrates.

In addition to the above ubiquitin dependent pathway and LC3 binding mediated mitochondrial autophagy, there are some damaged mitochondria that can also be recognized by LC3 adapter in a ubiquitin independent manner. These LC3 receptors are located in mitochondria, which can directly bind to LC3 and transfer damaged mitochondria to lysosomes for degradation. These receptors include BNIP3, Nix, Fundc1, PHB2 147-149.

### Mitophagy in DCM

The changes in mitophagy during DCM were evaluated using the mito-Keima assay in cardiomyocytes from GFP-LC3 mice fed a high-fat diet 150. Mitophagy was significantly induced after three weeks and further increased after two months of high-fat-diet treatment, accompanied by an increased end-diastolic pressure-volume relationship, lipid accumulation and cardiac hypertrophy 150. Knocking out *Parkin* partially suppressed cardiomyocyte mitophagy, elevated lipid accumulation and worsened diastolic dysfunction in response to high-fat-diet feeding 150. These data suggested that mitophagy is enhanced in the early stage of DCM. Although mitophagy is cardioprotective, endogenous mitophagy fails to prevent the progression of DCM. At the molecular level, mitophagy induction has been described as an adaptive response to increased fatty acid oxidation in the heart, because Parkin expression seems to correlate with the levels of acetyl CoA carboxylase 2, a regulator of long-chain fatty acid transport into mitochondria 151.

Using a novel dual-fluorescent mitophagy reporter (mt-Rosella), Kobayashi et al. traced dysfunctional mitochondria that were degraded in lysosomes, and found that chronic hyperglycemic stress impaired mitophagy in heart tissues from type-1 diabetic mice 152. In another study, mice were fed a high-fat diet for 10 weeks, and changes in FUNDC1-dependent mitophagy were monitored in the early stage of DCM 153. FUNDC1 expression was slightly elevated and therefore mitophagy was moderately enhanced in diabetic heart tissues 153. However, ablation of *FUNDC1* exacerbated myocardial inflammation, oxidative stress and cardiomyocyte apoptosis, suggesting that FUNDC1-dependent mitophagy is a defensive program, despite its failure to halt the progression of DCM 153.

Mitochondrial morphological changes in cardiomyocytes from diabetic mice were observed using electron microscopy 154. Most of the cardiomyocytes contained dissociated mitochondria that lacked typical contacts, and some of the mitochondria exhibited complete destruction of the cristae, “watering” of the matrix, and remains that resembled large vacuoles 154, 155. Expanded sarcoplasmic reticular profiles and glycogen accumulation were often recorded, and the contractile apparatus of the cardiomyocytes exhibited displaced myofibrils and tortuous Z-disks that were mismatched between adjacent myofibrils 154. However, activation of Parkin-induced mitophagy partly reversed the ultrastructural changes in cardiomyocyte mitochondria and prevented myocardial tissue disorganization 154. These findings confirmed that enhanced mitophagy, rather than basal mitophagy, is needed to sustain mitochondrial structure and myocardial homeostasis 156.

The protective mechanisms of increased mitophagy have been widely reported. Mitophagy was shown to attenuate mitochondria-induced cardiomyocyte apoptosis by reducing OMM hyperpermeability, cytochrome c release and oxidative stress 157. In addition to inhibiting apoptosis, mitophagy was found to repress cardiac ferroptosis, necroptosis and lipid peroxidation 153. Activation of Parkin-induced mitophagy maintained the mitochondrial membrane potential, normalized mitochondrial energy metabolism and balanced the redox response in the heart 158. During the development of DCM, mitophagy enhanced cardiomyocyte mitochondrial regeneration and biogenesis 159 and normalized the cardiac mitochondrial morphology and bioenergetics 160. By improving mitochondrial homeostasis 161, mitophagy was found to reduce lipid accumulation and increase both diastolic function (end-diastolic pressure-volume relationship) and systolic function (end-systolic pressure-volume relationship) in diabetic hearts 150. Moreover, the activation of mitophagy reduced body weight, improved hyperglycemic control, inhibited dyslipidemia, attenuated myocardial fibrosis and suppressed the inflammatory response (as evidenced by reduced transforming growth factor β1, hydroxyproline and brain natriuretic peptide levels) in the hearts of diabetic rats 162.

Targeted pharmacological approaches to activate mitophagy may be useful clinical therapeutic strategies to retard the progression and/or improve the prognosis of DCM (Table 4). The US Food and Drug Association has approved the anti-diabetic drug empagliflozin for the treatment of HF, regardless of diabetes status 163. Empagliflozin was reported to prevent HF by increasing the autophagic vacuole number and reducing myocardial fibrosis in diabetic hearts 164, 165. Liraglutide, a glucagon‑like peptide‑1 receptor agonist that has been used to treat diabetes and obesity, was shown to activate Sirt1, a protein deacetylase that depends on both adenosine monophosphate-activated protein kinase (AMPK) and NAD 158. Liraglutide thereby increased mitophagy activity in diabetic hearts; however, deletion of *Parkin* significantly abrogated these cardioprotective effects 158.

Melatonin, a regulator of the biological clock, was reported to increase the number of typical autophagosomes engulfing mitochondria in the heart, thus preventing hyperglycemia-induced cardiac remodeling during DCM 166; however, knocking out *Parkin* partly compromised these beneficial effects 160. In the hearts of *db/db* mice, the gasotransmitter hydrogen sulfide facilitated Parkin translocation onto mitochondria, thereby promoting mitophagy, improving cardiac function, reducing mitochondrial fragmentation, enhancing mitochondrial respiratory chain activity and suppressing mitochondrial apoptosis 167. Alisporivir, a non-immunosuppressive cyclosporin derivative and selective inhibitor of the mPTP, exerted cardioprotective effects by upregulating *PINK1* and *Parkin* mRNA expression in the heart tissues of diabetic mice, thus inducing mitophagy 154.

The D-β-hydroxybutyrate-(R)-1,3 butanediol monoester (ketone ester) diet was reported as a non-pharmacological approach to enhancing cardiac mitophagy 168. The ketone ester diet enhanced the resistance of mitochondria to oxidative stress, inhibited mPTP opening and increased mitochondrial succinyl-CoA:3-oxoacid-CoA transferase expression in cardiomyocytes 168. In addition, the ketone ester diet improved cytosolic E3 ubiquitin ligase translocation onto mitochondria and reinforced LC3-induced autophagosome formation in cardiomyocytes, leading to better cardiac systolic and diastolic function in mice with type-2 diabetes mellitus 168.

## Conclusions and perspectives

**Figure 4:**
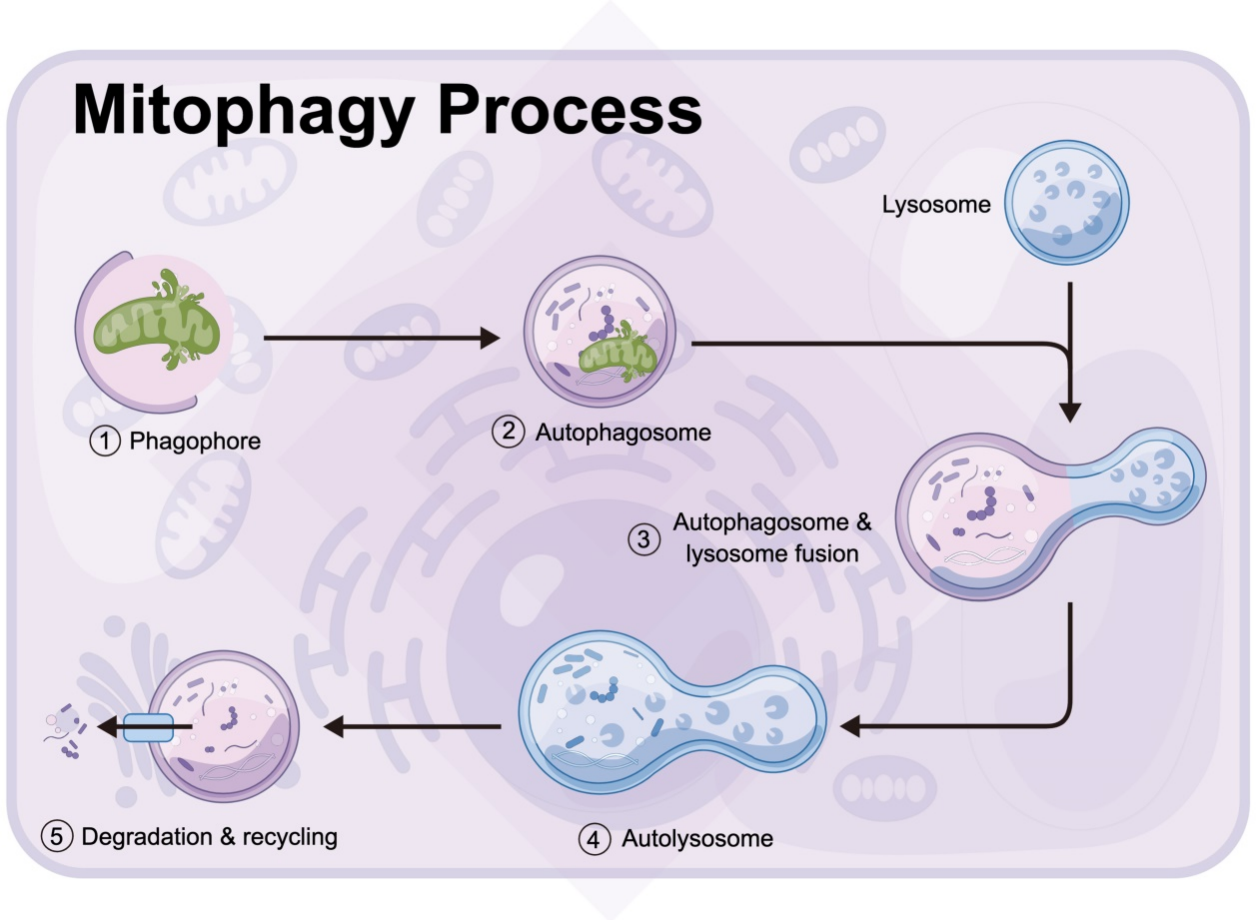
** Physiological MQC is an endogenous defense program that restores the mitochondrial integrity and homeostasis in response to mitochondrial damage; however, hyperglycemia compromises this protective mechanism.** Mitochondrial fission is overactivated in diabetic hearts, while fusion is markedly inhibited, resulting in extensive mitochondrial fragmentation. Under physiological conditions, mitophagy can engulf fragmented mitochondria; however, this process is inhibited under high-glucose conditions, so dysfunctional mitochondria accumulate within cardiomyocytes. Likewise, mitochondrial biogenesis can regenerate or replicate mitochondria, but hyperglycemia suppresses this process by inhibiting AMPK/PGC-1α. When MQC is blunted, mitochondrial dysfunction cannot be rectified, so the mitochondrial quality and quantity are further diminished.

The quality of mitochondria is maintained through the synthesis of new mitochondria, fusion and division, and the elimination of damaged mitochondria by mitophagy (Figure 4). With the increase of age, the changes of mitochondrion division and fusion process and the inhibition of mitophagy will lead to the decrease of mitochondrial biogenesis and the damage to mitochondrial clearance, which contributes to a series of metabolic disorders and pathophysiological diseases. In this review, we briefly discuss the roles of mitochondrial dynamics and mitophagy in regulating DCM. Meanwhile, the potential targeted therapies against mitochondrial dysfunction are also introduced in this review. However, it requires more attention to describe the detailed action afforded by MQC in DCM. Besides, new therapeutic approaches with a focus on MQC in the setting of DCM are the next clinical issue in the world.

## Figures and Tables

**Table 1:** Proposed classification of DCM

Stage of DCM^a^	Clinical phenotype
Stage I	Diastolic dysfunction with normal ejection fraction
Stage II	Combined systolic and diastolic dysfunction
Stage III	Systolic and diastolic dysfunction with microvascular disease/coronary atherosclerosis without obstructive coronary heart disease
Stage IV	Clinically overt ischemia/infarct causing HF

^a^Excluding coronary heart disease, valvular disease and uncontrolled hypertension.

**Table 2:** Summary of metabolic processes involved in the pathophysiology of DCM

Pathological mechanism	Pathophysiological pathway	Structural change	Functional alteration
Deranged Ca^2+^ homeostasis	Calcium leak from ryanodine receptor; Reduced sarcolemmal elimination of Ca^2+^; Prolonged Ca^2+^ transients	Mitochondrial leakage of toxic proteins; Myocardial cytotoxicity	Prolonged diastolic relaxation time; Myocardial stiffness; Impaired relaxation
Abnormal fatty acid metabolism	Increased systemic lipolysis; Loss of metabolic flexibility; Increased utilization of free fatty acids	Cardiac steatosis; Lipotoxicity; Myocyte apoptosis	Increased O_2_ consumption; Pathologic cardiac remodeling; Systolic dysfunction
Hyperglycemia	Activation of protein kinase C pathways; Production of free radicals	Myocardial necrosis; Dystrophic calcification	Myocardial fibrosis; LV hypertrophy; Diastolic dysfunction
Myocardial fibrosis	Transforming growth factor-β; Matrix metalloproteinase-2; Smooth muscle actin	Interstitial fibrosis; LV hypertrophy; Intimal thickening of microvasculature	Diastolic dysfunction; Systolic dysfunction
AGE/RAGE	Janus kinase pathway; MAPK activation	Cross-linking of extracellular matrix; Reduction of myocardial compliance Myocardial fibrosis	Prolonged isovolumetric relaxation time; Elevated LV end-diastolic diameter
ROS	Diacylglycerol Protein kinase C; NADPH-oxidase pathway	Oxidative myocardial injury; Mitochondrial damage Cardiac fibrosis	Myocardial stiffness; Diastolic dysfunction
Inflammation	NF-κB; Tumor necrosis factor-a; Interleukin-6	Inflammatory myocardial injury	Systolic dysfunction
Cardiac autonomic neuropathy	Hyperadrenergic state; Increased activation of b-receptors and RAAS	Interstitial fibrosis	Diastolic dysfunction
Altered protein homeostasis	Impaired ubiquitin proteasome system	Proteotoxicity; Myocardial cell damage	Pathological remodeling in diabetic hearts of animals
Microvascular dysfunction	Upregulation of vascular endothelial growth factor pathway	Fibrosis of capillaries	Impaired myocardial functional reserve

RAGE: AGE-specific receptor, NF-κB: nuclear factor kappa-light-chain-enhancer of activated B cells, RAAS: Renin-angiotensin-aldosterone system.

**Table 3:** Upstream signals of mitochondrial dynamics in the setting of DCM

Upstream regulator	Mechanism	Reference
Tom70	Tom70 enhances high-glucose and high-fat treatment-induced mitochondrial superoxide production, resulting in Drp1-induced mitochondrial fission.	121
Cyclin C	Cyclin C translocates to the cytoplasm and binds to cyclin-dependent kinase 1 to promote Drp1 phosphorylation at Ser616.	122
Sirt1	Sirt1 deficiency promotes Akt activation, thus increasing Drp1 activity, culminating in excessive mitochondrial fission and ROS production.	123
Estrogen	Estrogen upregulates Drp1 and downregulates Mfn2 in diabetic rats.	124
Gp78	The Gp78-ubiquitin proteasome system promotes the ubiquitination of Mfn1/2.	125
Insulin	Insulin treatment increases OPA1 protein levels, promotes mitochondrial fusion, increases the mitochondrial membrane potential and elevates both intracellular ATP production and oxygen consumption in cardiomyocytes.	126
Norepinephrine	Norepinephrine acts through α1-adrenergic receptors to increase cytoplasmic Ca^2+^ levels, thus activating calcineurin and promoting Drp1 migration to mitochondria.	127

**Table 4:** Targeted pharmacological or non-pharmacological therapeutic strategies to activate mitophagy for the treatment of DCM

Therapeutic strategy	Mechanism	Reference
Empagliflozin	Empagliflozin prevents diabetic HF by increasing the autophagic vacuole number in the heart, thus reducing myocardial fibrosis.	164
Ginseng Dingzhi Decoction	Ginseng Dingzhi Decoction activates mitophagy and thus ameliorates myocardial hypertrophy, heart function and mitochondrial homeostasis following high-glucose stimulation.	169
Liraglutide	Liraglutide activates the AMPK- and NAD‑dependent protein deacetylase Sirt1, thus increasing Parkin-induced mitophagy in diabetic hearts.	158
Melatonin	Melatonin increases the number of typical autophagosomes engulfing mitochondria through Parkin-induced mitophagy in diabetic hearts, thus reducing cardiac remodeling.	160
Hydrogen sulfide	Hydrogen sulfide facilitates Parkin translocation onto mitochondria and thus promotes mitophagy in the heart, ultimately reducing mitochondrial fragmentation, enhancing mitochondrial respiratory chain activity, suppressing mitochondrial apoptosis and improving cardiac function in *db/db* mice.	170
Alisporivir	Alisporivir upregulates *PINK1* and *Parkin* mRNA expression in the heart tissues of diabetic mice.	154
D-β-hydroxybutyrate-(R)-1,3 butanediol monoester (ketone ester) diet	A ketone ester diet improves cytosolic E3 ubiquitin ligase translocation onto mitochondria and reinforces LC3-induced autophagosome formation, thus enhancing cardiac systolic and diastolic function in animals with type-2 diabetes mellitus.	168
